# Evaluation of Metal‐Organic Framework MIL-89 nanoparticles toxicity on embryonic zebrafish development

**DOI:** 10.1016/j.toxrep.2022.04.016

**Published:** 2022-04-20

**Authors:** Dana E. Al-Ansari, Mashael Al-Badr, Zain Z. Zakaria, Nura Adam Mohamed, Gheyath K. Nasrallah, Huseyin C. Yalcin, Haissam Abou-Saleh

**Affiliations:** a Biological Science Program, Department of Biological and Environmental Sciences, College of Arts and Sciences, Qatar University, Doha, Qatar; b Division of Biological and Biomedical Sciences, College of Health and Life Sciences, Hamad Bin Khalifa University, Qatar Foundation, Doha, Qatar; c Biomedical Research Center, Qatar University, Doha, Qatar; d College of Health Sciences, Department of Biomedical Sciences, Qatar University, Doha, Qatar

**Keywords:** Danio rerio, Larvae, Development, Organ toxicity, Nanotechnology

## Abstract

Metal-Organic Framework MIL-89 nanoparticles garnered remarkable attention for their widespread use in technological applications. However, the impact of these nanomaterials on human and environmental health is still limited, and concerns regarding the potential risk of exposure during manipulation is constantly rising. Therefore, the extensive use of nanomaterials in the medical field necessitates a comprehensive assessment of their safety and interaction with different tissues of the body system. In this study, we evaluated the systemic toxicity of nanoMIL-89 using Zebrafish embryos as a model system to determine the acute developmental effect. Zebrafish embryos were exposed to a range of nanoMIL-89 concentrations (1 – 300 µM) at 4 h post-fertilization (hpf) for up to 120 hpf. The viability and hatching rate were evaluated at 24–72 hpf, whereas the cardiac function was assessed at 72 and 96 hpf, and the neurodevelopment and hepatic steatosis at 120 hpf. Our study shows that nanoMIL-89 exerted no developmental toxicity on zebrafish embryos at low concentrations (1–10 µM). However, the hatching time and heart development were affected at high concentrations of nanoMIL-89 (> 30 µM**).** Our findings add novel information into the available data about the in vivo toxicity of nanoMIL-89 and demonstrate its innocuity and safe use in biological, environmental, and medical applications.

## Highlights

• NanoMIL-89 is not lethal for zebrafish embryos.
• High concentrations of nanoMIL-89 alter the heart development and delay the hatching time in zebrafish embryos.
• Low concentrations of nanoMIL-89 showed no significant organ specific toxicity effects on zebrafish embryos.

## Graphical Abstract

**Figure:**
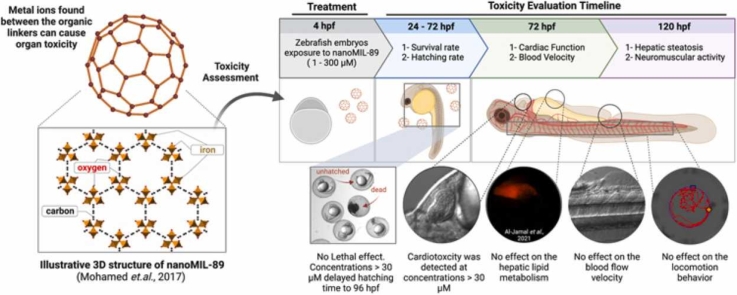

## 1 Introduction

Nanoparticles have recognized tremendous applications in many fields such as water purification, aquaculture development and sustainability, bioengineering, molecular imaging, drug delivery, and disease detection and treatment [1], [2], [3], [4]; In addition, nanoparticles have shown to possess phytoremediation, antibacterial, and anti-pesticide effects which make them a prominent tool in applied ecology [5], [6].

Material Institute Lavoisier 89 nanoparticles (nanoMIL-89), a subclass of Metal-Organic Frameworks (MOFs), have been extensively studied for their preeminent porous crystalline structure, large surface area, and high conformational flexibility due to their metal (iron) oxide core and organic linkers [7]. These structural properties made nanoMIL-89 salient vehicle carriers for variant drugs, proteins, nucleic acids, and bioactive molecules [8], [9], [10], [11], [12]. However, despite the advantageous features of nanoMIL-89, they may represent a significant risk of toxicity, which could be attributed to leached iron metal ions leading to high stress in cells, tissue and organ damage, or developmental alterations that cannot be depicted from in vitro studies [13], [14]. Generally, MOFs toxicity depends on the cell type, concentration, size, degradation rate, structure, nature of functionalized surface and solubility [13], [14]. Therefore, screening for teratogenicity of these particles and detailed characterization of their toxic effects in vivo is critical before their use for water treatment, aquaculture, biological, and theragnostic applications.

*Danio rerio* or Zebrafish (ZF) is a small vertebrate used as a model organism for various development and disease studies [15], [16] as well in toxicological applications [17], [18]. The ZF rapid embryonic maturation allows the evaluation of the complete development process of its embryo before it becomes fully self-sustained (approximately 5 days post-fertilization [19]). In addition, ZF shares a high degree (~70%) of genetic similarity, functional homology, and physiological and developmental processes with humans [15], [20]. Several studies have used ZF embryos to assess the toxicity of a wide range of nanoparticles, including iron-oxide [21], chistosan [6], [22], gold [23], silver [24], silica [25], as well as other subclasses of MOFs [14], [26].

We have previously synthesized and characterized a nano-formulation of MIL-89 and shown that at low concentrations (< 30 µg/mL) nanoMIL-89 has no harmful effects upon cellular internalization in a range of cell lines, including macrophages, epithelial, endothelial cells and smooth muscle cells [11], [12], [27]. However, the in vivo effects of nanoMIL-89 and their association with environmental health risks are unexplored [27].

In this study, we aim to unravel the adverse effects of nanoMIL-89, by conducting a set of organ-specific toxicity assays (cardiac, neuromuscular, and hepatic). We tested a range of nanoMIL-89 concentrations (1–300 µM) on ZF embryos following the Organization for Economic Cooperation and Development (OECD) guidelines for chemical toxicity assessments.

## 2 Material and methods

### 2.1 NanoMIL-89 preparation and concentrations

In this study, we used < 150 nm sized nanoMIL-89, which consists of an iron-based core-shell (iron (III) chloride hexahydrate) coordinated by organic linkers (*trans-trans* muconic acid) [12]*.* NanoMIL-89 were prepared and characterized using powder X-ray diffraction (PXRD) and dynamic light scattering (DLS) as previously described by our group [12], [27]*.* Briefly, iron (III) chloride hexahydrate (FeCl_3_. 6 H_2_O) (molecular weight (MW)= 270.3; 1 mmol) was mixed with trans-trans muconic acid (MW=142.1; 1 mmol) in absolute ethanol, then heated for 25 h at 100 °C in a Parr reactor. The precipitates were then retrieved by centrifugation and purified by serial washes in deionized water, followed by air drying to recover MIL-89 [27]. The nanoMIL-89 concentrations used in this study followed a half-log pattern (1, 3, 10, 30, 100, and 300 µM) as recommended by the OECD guidlines [28]. NanoMIL-89 were resuspended in E3 egg or N-Phenylthiourea (PTU) medium containing 0.1% Dimethyl sulfoxide (DMSO) and sonicated in an ultrasonic bath for 10 min to minimize any nanoparticles aggregation [14]. PTU medium was used in Prussian Blue staining, cardiac and hepatic assessment experiments, to impede the pigment formation in ZF embryos (AB strain) for better organs visualization under the microscope.

### 2.2 Zebrafish embryo culture

The wild-type ZF (AB strain) were maintained at 28 °C water temperature with a photoperiod of 14 h light and 10 h dark cycle in an environmentally controlled lab at the Biomedical Research Center (Qatar University, Doha, Qatar). On day 0, the healthy fertilized eggs were collected, rinsed, and placed in a petri-dish with E3 egg medium. At 4 h post-fertilization (hpf), the healthy fertilized eggs were collected, and the unfertilized or unhealthy eggs were discarded. The healthy embryos were then seeded in either E3 egg or PTU water mediums depending on the conducted assay. All experiments were performed at different endpoints from 24 hpf to 120 hpf as per the national and international regulations provided by Qatar University Institutional Animal Care and Use Committee (QU-IACUC) and the Department of Research at the Ministry of Public Health, Doha, Qatar [29]. At the end of each experiment, ZF embryos were euthanized with an overdose of tricaine methane-sulfonate (TMS, also known as MS-222), followed by rapid freezing as per the American Veterinary Medical Association guidelines for the euthanasia of animals [30].

### 2.3 NanoMIL-89 Prussian Blue staining

The internalization of nanoMIL-89 in ZF embryos was assessed using Prussian Blue to demonstrate iron (Fe) deposition content of the nanoparticles within the tissues at 120 hpf. For this experiment, 20 ZF embryos (4 hpf) were seeded in 6 well-plates for each experimental group. ZF embryos were exposed to 2 mL of nanoMIL-89 suspension (300 µM), PTU medium (control), PTU with 0.1% DMSO (negative control; NC) and 100 µM of ferric ammonium citrate (FAC) for Fe staining (positive control; PC). Prussian Blue staining was performed as per the manufacturer’s protocol (Sigma-Aldrich, St. Louis, Missouri, United States). Briefly, the working solution of the Prussian Blue staining was prepared by mixing a 1:1 vol of hydrochloric acid and potassium ferrocyanide solutions, and the pararosaniline solution was prepared as per the manufacturer’s protocol. Thereafter, 12 ZF larvae of each treatment group were collected at 120 hpf into a 1.5 mL Eppendorf tube and rinsed twice with deionized water before staining. An amount of 1 mL of the iron staining solution was added to each experimental group and incubated for 13 min at room temperature. The larvae were then rinsed with deionized water and incubated for 4 min with 1 mL of the counterstain pararosaniline solution. The dehydration of larvae was then done through a series of incubations in increasing alcohol concentrations as indicated in the manufacturer protocol. After staining, ZF larvae were examined under ZIESS Stemi-508-stereomicroscope using a depression glass slide.

### 2.4 Acute toxicity assays

The toxicity of nanoMIL-89 was investigated using acute toxicity assays that adapt the OECD guidelines for chemical toxicity assessments (Nº 203 and 236), which were modified for nanoparticle toxicology [31], [32]. At 4 hpf, healthy ZF embryos were seeded into 96 well-plate (1 embryo/ well) with a total of 24 embryos for each concentration. ZF embryos were then treated with 200 µL of increased concentrations (1, 3, 10, 30, 100, and 300 µM) of nanoMIL-89 suspensions (dissolved in E3 egg water medium) followed by an incubation at 28 °C for up to 120 hpf. ZF embryos treated with 0.1% DMSO were used as NC, whereas 3% ethanol (EtOH) treated group was used as PC for lethality [33]. The viability of ZF embryos was assessed using a standard dissecting microscope and recorded at three time-point intervals (24, 48, and 72 hpf). Meanwhile, the effect of nanoMIL-89 on the hatching time of treated ZF embryos was assessed at 72 and 96 hpf. The viability and the hatching rate were calculated as the ratio of viable or hatched embryos to the total viable embryos x100, respectively.

### 2.5 Heart muscle and blood flow imaging

The effects of nanoMIL-89 on the cardiac function and development of the ZF embryos were assessed by imaging the heart and blood vessels at 72 hpf post embryo hatching. First, unhatched embryos were gently force hatched using forceps to unleash the larvae from their chorions. Then, the imaging of the heart muscle and blood flow video recordings were done for 5 embryos from each experimental group as previously described [34]. Briefly, ZF larvae were moved upon imaging into a depression glass slide in 3% methylcellulose, mixed with egg water to hinder their movement, and oriented in a lateral view, facing left with their yolk sac facing up. The imaging was done at a magnification of 100X for 5 s at 100 frames per second (fps) for the heart muscle contraction. Meanwhile, for the blood flow imaging, two major blood vessels, the dorsal aorta (DA), and posterior cardinal vein (PCV) were imaged at 100X for 10 s at 100 fps [34]. The video recordings were done using Zeiss SteREO Discovery V12 Microscope equipped with Hamamatsu Orca Flash high-speed camera and HCImage software V4.4.1.0 workstation (Hamamatsu Photonics, Japan).

### 2.6 Cardiac function and hemodynamics assessment

To quantitate the physiological parameters of the cardiac function and hemodynamics, the videos were analyzed using a quantitative structural analysis of 2D images [35], [36]. Briefly, using ImageJ software V1.51, the video recordings were first converted into fps image sequence. Then, the systolic state (the frame where the final forward flow of blood from the outflow tract is detected) and the diastolic state (the frame where the final flow of blood into the ventricles is detected) were randomly selected for each embryo from different treatments [37], [38]. The areas, length, and axes of the ventricular cavity and myocardial wall dimensions were then measured, at both the diastolic and systolic frames [37], [38]. After collecting the structural measurements and parameters from ImageJ, the cardiac output (CO), stroke volume (SV), ejection fraction (EF), diastolic and systolic ventricular volumes as well as heartbeats per minute were calculated using optimized equations of ventricular performance [34], [35], [36], [37]. Finally, the blood flow velocity in the DA and PCV was measured by tracking and analyzing the red blood cells movement via image analysis algorithms in Viewpoint (version 3.4.4, Lyon, France) [34], [39].

### 2.7 Cardiotoxicity assay

To further examine the effects of nanoMIL-89 on cardiotoxicity, the mRNA expression of the heart failure marker, atrial natriuretic peptide (*ANP*), was measured in real-time quantitative-PCR (RT q-PCR). In this experiment, 0.625 µM Aristolochic acid (AA) was used as PC to induce heart failure as described in previous studies [40], [41]. The total RNA of the nanoMIL-89 treated ZF larvae (10, 100, and 300 µM) along with the NC, and PC were extracted at 72 hpf [42]. The cDNA synthesis was then performed using the High-Capacity cDNA Reverse Transcription Superscript™ IV VILO™ Master Mix kit (ThermoFisher Scientific, Waltham, Massachusetts, United States), as per the manufacturer's instructions. Following the cDNA synthesis, the RT q-PCR was carried out using TaqMan® Fast Advanced Master Mix (Applied Biosystems, Waltham, Massachusetts, United States) and TaqMan primers against the myocardial stress predictive marker gene, *ANP*, and the housekeeping gene β2-Microglobulin (*B2M*) (APGZVJD, Catalog# 3251371 and Dr03432699_m1, Catalog# 4351372, respectively) (ThermoFisher Scientific, Waltham, Massachusetts, United States). Then, the mRNA expression signal was read using RT-qPCR (QuantStudio™ 6 Flex RT-qPCR System), and the relative quantity calculation was done using the 2^-△△CT^ method [43]*,* with the fold change being calculated in reference to the expression of the housekeeping gene, *B2M*.

### 2.8 Neuromuscular toxicity evaluation

The locomotion assay was performed to determine the effects of nanoMIL-89 on the development of ZF embryos nervous systems by assessing the neuromuscular activity. At 4 hpf, healthy ZF embryos were seeded in 200 µL E3 egg water medium in a 96 well-plate (1 embryo/well) with a total of 12 embryos for each concentration. In this experiment, 100 µM of 1-Methyl-4-phenyl-1,2,3,6-tetrahydropyridine hydrochloride (MPTP), which induces mitochondria fragmentation in ZF neurons, was used as PC for neurotoxicity [44], [45]. The locomotion assessment was done at 120 hpf, using ViewPoint ZebraBox technology (ViewPoint Life Sciences Lyon, France) as described in our previous work*.* [6], [34]. Briefly, treated ZF embryos were placed in a 96-well plate in the system chamber illuminated with white light for a calibration period of 20 min at 28 °C. After that, the ZF embryos movement was measured every 5 min in alternating dark/light cycles, where 10 min darkness was followed by 10 min of light for three cycles (total of 50 min). The average distance per 5 min and the total distance traveled were calculated per experimental group.

### 2.9 Detection of hepatic steatosis

The effect of nanoMIL-89 on the hepatic lipid metabolism and accumulation in treated ZF embryos was evaluated through yolk retention examination using Oil Red O (ORO) staining (Sigma-Aldrich, St. Louis, Missouri, United States). ORO staining is a fat-soluble dye that stains lipids and triglycerides explicitly. The working stain solution of ORO was prepared as described previously [46]. In brief, 0.035 g of ORO dry powder was resuspended in 7 mL of absolute isopropanol and incubated stirring overnight at room temperature. The working solution of the ORO stain was then prepared by mixing it with 10% isopropanol in a 1:1 ratio. In this experiment, ZF embryos were treated with nanoMIL-89 as mentioned in the previous sections, and 1% EtOH was used as PC for yolk retention, which was introduced to ZF embryos at 96 hpf [47]. At 120 hpf, the treated ZF larvae were first collected in 1.5 mL Eppendorf tubes and washed from the PTU medium with 60% isopropanol. The sample size of this experiment is 15 larvae per treatment pooled in one tube, with each treatment having three replicates. An amount of 1 mL of ORO staining working solution was then added to the treated ZF larvae and incubated for 75 min at room temperature. The stained larvae were then rinsed with 60% isopropanol and washed with 1% PBS. To extract the ORO from the stained larvae, 1% PBS was replaced with 250 µL of 4% ethanol in absolute isopropanol, followed by overnight incubation at room temperature. The absorbance of the extracted ORO stain was then read on a Tecan GENIOS Pro-200 at 495 nm by pipetting an amount of 200 µL of each sample tube into a 96-well plate.

### 2.10 Statistical analysis

Results are presented as mean ± standard error of the mean (SEM). The treated groups were compared to control (ZF in normal medium) using one-way analysis of variance (ANOVA) followed by Dunnett test. Significance is presented as (*) = p < 0.05; (**) = p < 0.01; (***) = p < 0.001 and (****) p < 0.0001. All statistical tests used to analyze the presented data are reported in the corresponding figure legend.

## 3 Results and discussion

In this study, we examined the acute toxicity of nanoMIL-89 on ZF embryos, which are recognized by the National Institute of Environmental Health Science (NIEHS) and the National Institutes of Health (NIH) as an exemplary model system in environmental toxicity studies and for exploring human diseases [48], [49], [50]. A study by Ruyra et al.*,* has previously evaluated the toxicity of different classes of MOFs in ZF embryos, such as nanoMIL-100, nanoMIL-101, MOF-74, UIO-66, and UIO −67, which were tested at a range of concentrations of 25 – 200 µM [14]. However, the evaluation was limited to the cumulative survival percentage and hatching rate. Therefore, in this study, we carried out organ-specific toxicity assays that are designed to reflect the effect of nanoMIL-89 on different organ systems, at a similar but broader range of nanoMIL-89 concentrations (1–300 µM). These include the cardiac, neuromuscular, and hepatic functions. The assays target directly or indirectly specific developmental biomarkers, which in turn reflect the toxicological effects of nanoMIL-89. For instance, in the cardiotoxicity evaluation, we assessed *ANP* gene expression, a reliable gold standard diagnostic biomarker in heart failure [51], [52]. Moreover, the locomotion assay was used to assess the neurogenesis and myogenesis processes in ZF embryos [53].

### 3.1 Biodistribution of nanoMIL-89 in ZF embryos

**Fig. 1:**
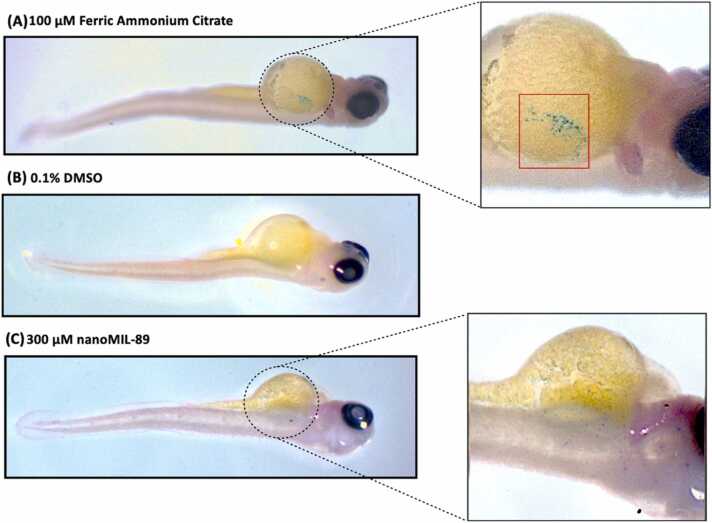
Prussian Blue iron stain of Zebrafish embryos at 120 hpf. Zebrafish embryos were treated at 4 hpf with 300 µM of nanoMIL-89, 0.1% DMSO used as a negative control (NC) and 100 µM Ferric ammonium citrate (FAC) used as a positive control. At 120-hpf post treatment, zebrafish embryos were collected and stained for iron using Prussian Blue stain. Stereomicroscopic images show (A) positive control FAC treated, (B) 0.1% DMSO treated (NC) and (C) 300 µM nanoMIL-89 treated larvae. The iron content of nanoMIL-89 aggregations was stained in blue. Red box indicates iron accumulation resulted from FAC.

Nanoparticles have shown the ability to penetrate living systems and deposit in various tissues [54]. Therefore, assessing nanoMIL-89 uptake is capital for understanding their distribution and biological effects within the ZF embryos system [54]. The internalization of nanoMIL-89 in ZF was examined using Prussian Blue, which stains the ferric iron in nanoMIL-89. At 120 hpf, ZF embryos were collected, stained, and imaged using ZIESS stemi-508-stereomicroscope (Fig. 1). Traces of iron were detected in ZF larvae treated with 100 µM of FAC (PC) whereas untreated ZF larvae showed no traces of iron staining (Fig. 1A and B). Interestingly, iron traces of nanoMIL-89 microaggregates were shown to be thoroughly distributed around the ZF embryos’ body organs, including the heart muscle, blood vessels, and the yolk sac of the treated ZF embryos, as indicated in Fig. 1C. Accordingly, the effect of nanoMIL-89 on neuromuscular, heart, and hepatic functions were assessed further in this study.

### 3.2 High concentrations of NanoMIL-89 delay the hatching time of ZF embryos

**Fig. 2:**
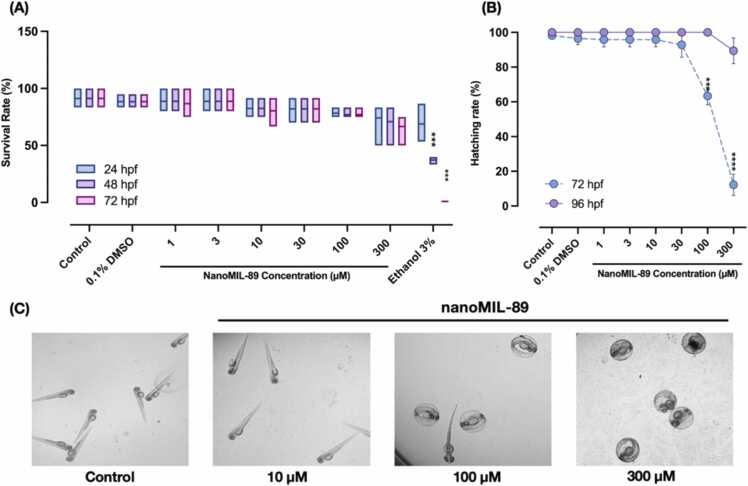
The survival and hatching rate of Zebrafish embryos post nanoMIL-89 treatment. (A) The viability of zebrafish embryos was assessed at 24, 48, 72 h post fertilization (hpf), post nanoMIL-89 treatment. Zebrafish embryos treated with 0.1% DMSO and 3% Ethanol represents the negative control and lethal positive control groups, respectively. (B) The hatching rate of zebrafish embryos was assessed at 72 and 96 hpf post nanoMIL-89 treatment. (C) Representative stereomicroscopic images of zebrafish embryos at 72 hpf treated with different nanoMIL-89 concentrations. Data are mean of ± SEM; n = 56, groups were compared using Ordinary One-way ANOVA to the control (*p < 0.05, ***p < 0.001 and ****p < 0.0001).

The effect of nanoMIL-89 on ZF embryos viability and hatching rate was assessed by exposing ZF embryos to different concentrations of nanoMIL-89 at 4 hpf. The viability was examined at different time points (24, 48, and 72 hpf). The percentage of cumulative survival rate of treated ZF embryos was calculated for increasing concentrations of nanoMIL-89 (1–300 µM), 0.1% DMSO (NC), and 3% EtOH (PC). The viability of ZF embryos was not affected by nanoMIL-89 treatment, as shown in Fig. 2A. Moreover, high concentrations of nanoMIL-89 (> 30 µM) showed moderate, but not significant, effect on the viability of ZF embryos (~66.7%). Noteworthy, at concentrations > 300 µM, nanoMIL-89 did not show any lethal effects on ZF embryos due to nanoparticles aggregations caused by medium oversaturation (data not shown). Hence, the LC50 of nanoMIL-89 was not determined.

The hatching rate of ZF embryos was evaluated at 72 hpf and expressed as the percentage of hatched embryos to the total viable embryos. The hatching rate for concentrations ≤ 30 µM was not affected by nanoMIL-89 (Fig. 2B). However, a significant dose-dependent delay in the hatching rate was observed with concentrations > 30 µM (Fig. 2C) with an average hatching rate of 63.3% and 12.2% for 100 µM and 300 µM nanoMIL-89 at 72 hpf, respectively. Nevertheless, the hatching rate was delayed to 96 hpf for the above concentrations (Fig. 2B). These findings are consistent with a previous study by Ruyra et al., reporting that Fe-based MOFs nanoparticles MIL-100 and MIL-101 did not exert any lethal effect on ZF embryos, even at high concentrations. Moreover, In the same study, MIL-101 and MIL-100 were shown to delay the hatching rate of zebrafish embryos at 200 µM [14].

Hatching is a crucial stage of ZF embryogenesis, and a delayed hatching rate indicates a stress response due to a possible disturbance in the anatomy or physiology of the ZF embryos development [55], [56]. In line with other studies, the organic linkers and iron oxide composites of MOFs nanoparticles can impact the activity of the chorionase enzyme and cause an osmotic disturbance, which in turn contributes to the delay of the ZF hatching process [21], [26], [57]. Moreover, the adherence and agglomeration of nanoMIL-89 on the chorion surface cause oxygen depletion and hypoxia, which may delay the hatching of ZF embryos and development [21], [58].

### 3.3 Low concentrations of nanoMIL-89 do not affect the hemodynamic functions and blood flow velocity

**Fig. 3:**
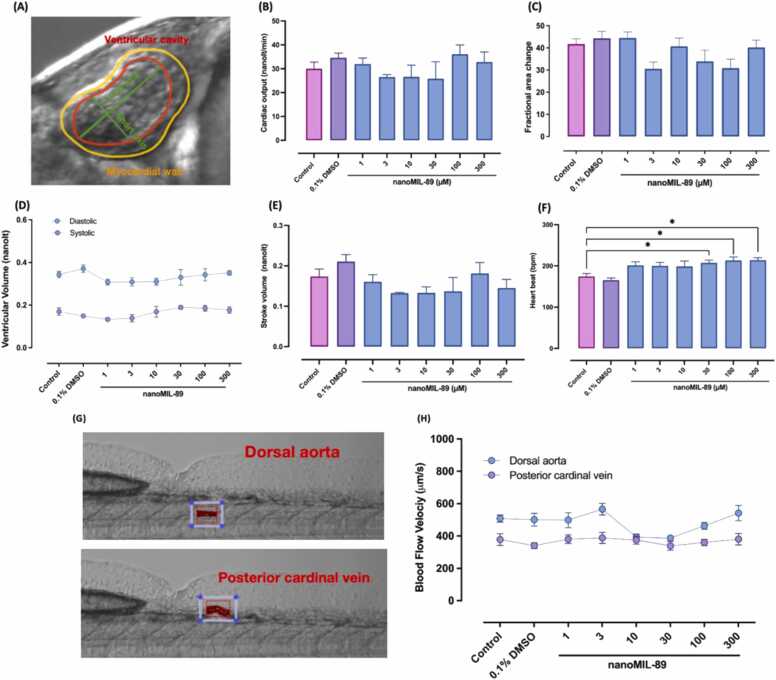
The Assessment of the Cardiac Function and Blood Flow Velocity Zebrafish Embryos at 72 hpf. The heart, dorsal aorta (DA) and the posterior cardinal vein (PCV) of the hatched zebrafish embryos treated with nanoMIL-89 was imaged using Zeiss SteREO Discovery V12 Microscope equipped with Hamamatsu Orca Flash high-speed camera at 100X at 100 frames per second. Using ImageJ software, (A) the ventricular cavity and myocardial wall were first determined to calculate and analyze the cardiac function and hemodynamics: (B) Cardiac output (nanolt/min); (C) Stroke volume (nanolt); (D) ventricular volume (nanolt); (E) Ejection Fraction (%) and (F) Heart beats per minute (bpm). (G) The blood flow velocity of wild-type and nanoMIL-89 treated zebrafish embryos were measured by tracking and analyzing the red blood cells movement in the DA and the PCV via image analysis algorithms in Viewpoint. (H) The blood flow velocity of the DA and PCV were plotted in respect to different concentration of nanoMIL-89. Data are mean ± SEM; n = 5, Ordinary One-way ANOVA was used to compare the treated groups to the control group. (*p < 0.05).

Cardiotoxicity is a major contributing factor for drugs and nanoparticles withdrawal from biological applications and clinical trials [18]. Multiple studies have utilized ZF embryos for cardiotoxicity assessments and teratogenic effect of nanoaprticles [6], [59], [60]. Zebrafish embryos' heart contractions initiate at 24 hpf, followed by the division of the looped heart into two chambers through the atrioventricular canal formation at 48 hpf [60]. By 72 hpf, the epicardium covers the myocardial surface, the cardiac tubercular formation is initiated and the ZF embryo has almost all major cardiac components [61], [62]. To evaluate the effect of nanoMIL-89 on the heart muscle function, several physiological parameters were determined in our assay, including the CO, SV, ventricular volume, EF heartbeats, and blood velocity. At 72 hpf, the heart muscle, DA, and PCV were imaged at a magnification of 100X and recorded at 100 fps. The dimensions of the ventricular cavity and myocardial wall of embryos’ hearts were first measured using ImageJ software V1.51 (Fig. 3A), followed by assessing the cardiac functions and hemodynamics (Fig. 3B–F). Although NanoMIL-89 did not significantly affect the CO, SV, ventricular volume, and EF functions, a minor significant dose-dependent increase was observed in the treated ZF embryos heartbeats (> 190 bpm). A similar effect was reported in a previous study by Rajesh et al., where iron oxide nanocomposites were shown to induce tachycardia in ZF embryos in a dose-dependent manner [57].

Besides the heart function, it is important to investigate the blood flow, and wall shear stress in the cardiovascular system of ZF embryos [39]. There are two major blood vessels in ZF embryos, the DA and the PCV. Blood velocity was assessed by tracking the RBCs motion within the major blood vessels, using Viewpoint as shown in Fig. 3G. No significant changes were observed in the blood flow velocity of the nanoMIL-89 treated ZF embryos compared to the control group (Fig. 3H). Hence, our data show that nanoMIL-89 do not exhibit a significant cardiotoxicity effect on embryonic ZF.

### 3.4 High concentrations of nanoMIL-89 slightly upregulate the expression levels of the heart failure marker ANP

**Fig. 4:**
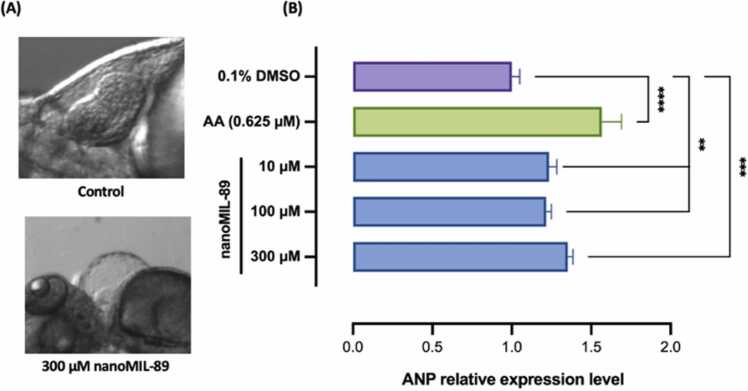
Quantitative Analysis of the mRNA Expression of ANP, Myocardial Stress Predicative Marker, in NanoMIL-89 Treated Zebrafish Embryos. (A) Microscopic images (100X) of Zebrafish Embryos (72 h post fertilization; hpf) treated with 300 µM nanoMIL-89 showing cardiovascular morphological defects compared to wild-type Zebrafish embryo. (B) The mRNA expression of the myocardial stress marker, atrial natriuretic peptide (ANP) in nanoMIL-89 treated zebrafish embryos were analyzed via real time quantitative-PCR (RT q-PCR) at 72-hpf. Zebrafish embryos treated with 0.625 µM Aristolochic acid (AA) represent the positive control. Data are mean ± SEM; n = 15 embryos with 3 replicates. Statistical analysis was done using Ordinary One-way ANOVA to compare ANP expressions to the negative control (0.1% DMSO), **p < 0.01, ***p < 0.001 and ****p < 0.0001.

Although nanoMIL-89 did not have any morphological abnormalities on ZF embryos at concentrations < 300 µM, heart deformations were detected in few embryos treated with 300 µM of nanoMIL-89 and hatched normally at 72 hpf (Fig. 4A). Therefore, to further investigate the cardiotoxicity effect of nanoMIL-89 on ZF embryos' heart development, an RT q-PCR of the myocardial stress marker *ANP* was carried out at 72 hpf. Compared to the NC, *ANP* mRNA expression levels showed a minor dose-dependent increase in treated ZF embryos, reaching a maximum of ~1.35 folds change at 300 µM of nanoMIL-89 (Fig. 4B). Meanwhile, the PC-treated group (AA, 0.625 µM) showed a more significant increase by 1.56 folds in *ANP* levels. The minor increase in the *ANP* levels can explain the morphological defects seen in 300 µM treated ZF embryos, as *ANP* is usually expressed in response to myocardial stress and heart failure [40], [63]. However, as reported in the literature, the expression of *ANP* is also proven to have a protective role in preserving the homeostatic function and reversing vascular remodeling, as reported in previous studies [64], [65], [66].

### 3.5 NanoMIL-89 does not alter the neurobehavioral activities of ZF embryos

**Fig. 5:**
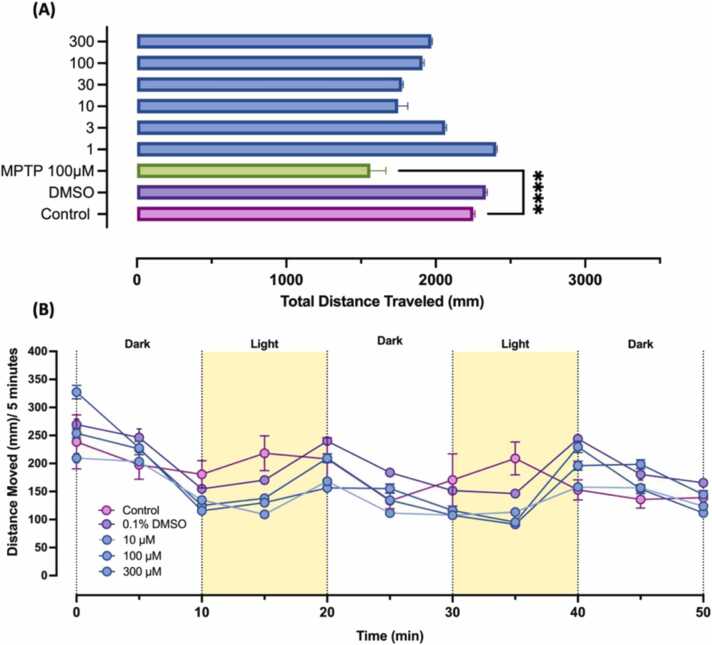
The Assessment of neurodevelopment toxicity of nanoMIL-89 at 120 h post fertilization (hpf) using locomotion assay. Zebrafish embryos were treated with different concentrations of nanoMIL-89, 100 µM of 1-Methyl-4-phenyl-1,2,3,6-tetrahydropyridine hydrochloride (MPTP) as a positive control and 0.1% DMSO as a negative control. At 120-hpf, the locomotion was assessed by measuring (A) the total distance traveled (mm) and (B) the average distance moved (mm) by zebrafish larvae per 5 min with an alternating light/dark cycles every 10 min, using ViewPoint software for a total of 50 min. Data are mean of ± SEM; n = 12, nonparametric One-way ANOVA was used to compare the total distance traveled of the treated groups to the control group (****p < 0.0001).

Among other model systems, ZF embryos have the advantage of behavioral connection with the nervous system. At 72 hpf, ZF embryos enter the larval stage, where their behaviors are exploited for different applications such as screening drugs with central nervous system effects [53]. Similarly, in our study, we are assessing the neurotoxicity of ZF embryos in response to nanoMIL-89 using the locomotion assay. The locomotion assay is a high-throughput method that is commonly used to identify neuroactive drugs [67]. In this assay, the ZF larvae are expected to respond to the dark/light transitions, with the activity being higher in the light phase and lower in the dark phase. At 120 hpf, the swimming behaviors and locomotion activity of nanoMIL-89 treated ZF embryos were evaluated using ViewPoint ZebraLab. The total distance traveled by ZF embryos was measured after 50 min of 10 min dark/light cycles. Compared to the control, nanoMIL-89 treated ZF embryos did not show significant changes in the total distance traveled (Fig. 5A). In contrast, the PC group (100 µM MTPT) showed a significant reduction in its locomotion activity, as observed in previous studies [44], [68]. Moreover, nanoMIL-89 treated ZF embryos showed normal locomotion behaviors during the dark/light phases similar to the control group (Fig. 5B), with the activity being higher during the light phase and lower during the dark phase [68]. This indicates the normal primary motor neuron innervation in the skeletal muscle during the ZF development via transcriptional control of master muscle development regulators [69]. Thus, no significant neuromuscular toxicity was observed in ZF embryos treated with nanoMIL-89.

### 3.6 NanoMIL-89 do not affect the hepatic lipid metabolism in ZF embryos

**Fig. 6:**
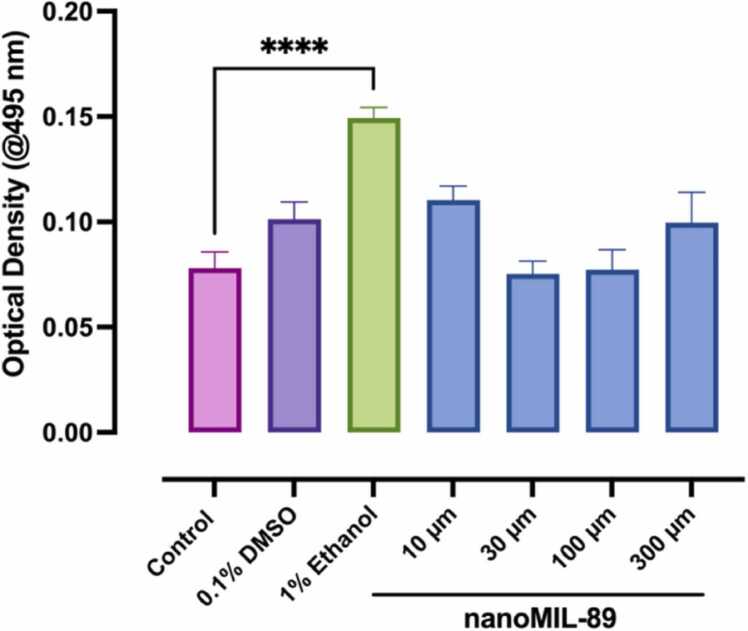
The optical density analysis of Oil Red O (ORO) stained embryos treated with nanoMIL-89. The yolk retention examination in treated zebrafish embryos was done by ORO staining at 120-hpf, followed measuring the absorbance of the extracted ORO stain using Tecan GENIOS Pro-200 at 495 nm. Zebrafish embryos treated with 1% Ethanol represent the positive control group. Data are mean ± SEM; n = 25 embryos with 6 replicates for ORO staining. Statistical analysis was done using Ordinary One-way ANOVA to compare the OD at 495 nm of treated zebrafish embryos to the control (****p < 0.0001).

Zebrafish embryos have been extensively used to evaluate the effect of drugs, chemicals, and molecules on the hepatic function through well-established functional assays [70], [71], [72], [73], as they highly conserve genetic pathways which underlay liver disease [74]. Around 70% of the ZF embryos yolk sac is composed of lipids, which are primarily metabolized by hepatocellular uptake [75], [76], [77]. An injury to the hepatocytes due to toxic substances and chemicals exposure, such as drugs, nanoparticles, and other toxins, can alter the hepatic lipid metabolism, leading to the accumulation of lipids in the liver or hepatic steatosis [75], [77]. Therefore, we indirectly evaluated the hepatic lipid metabolism function in response to nanoMIL-89 treatment by assessing the neutral lipid accumulation and triglyceride content. This was realized at 120 hpf, when the ZF embryo's liver is wholly developed and fully functional [70], [71], using ORO lipid-specific staining. The intravascular lipids of ZF embryos were quantified by extracting the ORO stain and measuring the absorbance at 495 nm. According to the optical density (OD) analysis (Fig. 6), ZF embryos treated with 1% EtOH (PC) showed a significant increase by more than 50% in lipids accumulation (0.146), which is consistent with previous studies [6], [78]. However, the OD values of nanoMIL-89 treated ZF embryos were not significantly different from the control group (Fig. 6). Thereby, no alteration in the hepatic function was detected upon ZF embryos incubation with nanoMIL-89.

## 4 Conclusion

For the first time, our study has comprehensively investigated the organ-specific toxicity of nanoMIL-89 and their in vivo systemic effects using ZF embryo model. Our results demonstrate that nanoMIL-89 has no significant lethal effects on ZF embryos at the used concentrations (0–300 µM). However, the hatching time of ZF embryos was significantly delayed at concentrations > 30 µM of nanoMIL-89. Meanwhile, the cardiac development was altered at concentrations > 100 µM. This was associated with a slight increase in the expression of the heart failure marker, *ANP*, suggesting possible harmful effects of nanoMIL-89 at high concentrations (> 30 µM). Meanwhile, concentrations ≤ 30 µM, nanoMIL-89 did not significantly affect the neuromuscular, cardiac, and hepatic lipid metabolism functions. Our data suggest that nanoMIL-89 is considered safe at relatively low concentrations (< 30 µM). Nevertheless, more organ-specific toxicity assays, such as kidney toxicity, and the screening of liver toxicity-specific markers (ALT, AST, and GSH) will be the object of future studies. We believe that our results add new insight into the in vivo effect of nanoMIL-89 and provide valuable information regarding their employment in biological and technological applications.

## Ethics approval and consent to participate

The experiments comply with the current policy of ZF research, stated by the research regulation department in the ministry of public health, Doha, Qatar [29]. The ZFET assays were carried out for up to 120 hpf, in accordance with OECD ethical standards [28].

## CRediT authorship contribution statement

**Dana E. Al-Ansari:** Conceptualization, Methodology, Visualization, Formal analysis, Validation, Investigation, Data curation, Writing – original draft and Writing – review & editing. **Mashael Al-Badr:** Visualization, Methodology, Formal analysis, Data curation, Visualization and Review and Editing. **Zain Z. Zakaria:** Visualization, Methodology, Formal analysis, Data curation, Writing – review & editing. **Nura Adam Mohamed:** Methodology, Formal analysis, Data curation, Visualization and Review and Editing. **Gheyath K. Nasrallah:** Methodology, Investigation, Data curation and Review and Editing; Huseyin C. Yalcin*: Methodology, Validation, Investigation, Resources, Co-supervision. Haissam Abou-Saleh*: Conceptualization, Methodology, Validation, Investigation, Resources, Data curation, Writing – review & editing, Supervision, Project Administration, Funding acquisition.

## Declaration of Competing Interest

The authors declare that they have no known competing financial interests or personal relationships that could have appeared to influence the work reported in this paper.
